# Photo-Fenton Degradation Process of Styrene in Nitrogen-Sealed Storage Tank

**DOI:** 10.3390/toxics11010026

**Published:** 2022-12-27

**Authors:** Yiqiang Zhao, Meng Liu, Xiaolong Xu, Chunxu Li, Jiaji Cheng, Zhimeng Wang, Dong Wang, Wenjuan Qu, Shaoxiang Li

**Affiliations:** 1 College of Environmental and Safety Engineering, Qingdao University of Science and Technology, Qingdao 266042, China; 2 Shandong Furixuanwei New Materials Technology Co., Ltd., Weifang 261000, China; 3 College of Mechanical and Electrical Engineering, Hohai University, Changzhou 213022, China

**Keywords:** styrene, UV/Fenton, nitrogen, VOCs, BAS-BP neural network

## Abstract

Using styrene as a proxy for VOCs, a new method was developed to remove styrene gas in nitrogen atmospheres. The effect on the styrene removal efficiency was explored by varying parameters within the continuum dynamic experimental setup, such as ferrous ion concentration, hydrogen peroxide concentration, and pH values. The by-products are quantized by a TOC analyzer. The optimal process conditions were hydrogen peroxide at 20 mmol/L, ferrous ions at 0.3 mmol/L and pH 3, resulting in an average styrene removal efficiency of 96.23%. In addition, in this study, we construct a BAS-BP neural network model with experimental data as a sample training set, which boosts the goodness-of-fit of the BP neural network and is able to tentatively predict styrene gas residuals for different front-end conditions.

## 1. Introduction

VOCs are the most toxic and react photochemically with airborne nitrogen oxides under light exposure to produce photochemical fumes that pose a hazard to human health and crop growth [1]. VOCs originate from a wide range of sources, including industrial production, automobile exhaust, and numerous others. The management of VOCs has received considerable attention in recent years [2,3].

China’s styrene industry has entered a period of rapid growth. It is the world’s fastest-growing country in styrene consumption and the world’s largest producer and consumer of styrene. Styrene gas is treated by combustion and activated carbon adsorption methods commonly used in industry [4,5,6]. Styrene needs to be stored in nitrogen-sealed tanks, and there is a high safety hazard if styrene gas is degraded by combustion in the tank area and increased economic costs if styrene gas is delivered to distant locations [7,8]; if the adsorption method is adopted to adsorb styrene gas, the internal structure of activated carbon will be clogged due to the self-polymerization effect of styrene, thereby reducing the adsorption efficiency and also the risk of autoignition [8]. Therefore, there is literature about different methods to degrade styrene gas, such as the biodegradation method and advanced oxidation method etc. [5,9]. Heitz et al. employed two nutritional forms of ammonia and nitrate to exclusively degrade styrene at low concentrations [5], and Kamila Koci et al., employed UV light combined with ozone/hydrogen peroxide to degrade 87% of styrene gas [9]. However, in the approach adopted by Kamila Koci et al., ozone was applied, and the scenario applied in this study was canned. In order to reduce the probability of a hazardous accident, this study will reduce the concentration of oxygen content during the reaction.

In this study, a novel UV/Fenton process for the degradation of styrene gas emitted from the breathing of a nitrogen-sealed tank size. In the laboratory, the high-purity nitrogen was used to blow off a washing cylinder containing styrene liquid. The concentration of the exhaust gas is controlled by changing the volume of styrene in the wash cylinder, and then the nitrogen flow containing styrene is introduced into the reaction for oxidation, i.e., the degradation of styrene with an elevated concentration (2000 mg/m^3^) by the UV/Fenton method was carried out under the nitrogen atmosphere. This technique used Fe^2+^ as the catalyst to decompose H_2_O_2_ to produce (•OH) [10], where the addition of ultraviolet light (UV) decreased the amount of Fe^2+^ and reduced the generation of iron sludge [11]. The Fenton reaction principle is as follows:

(1) $$H_{2}O_{2}+{\mathrm{Fe}}^{2+}\overset{}{\to }{\mathrm{OH}}^{-}+{\mathrm{OH}}^{•}+{\mathrm{Fe}}^{3+}$$

(2) $${\mathrm{Fe}}^{3+}+H_{2}O_{2}\overset{}{\to }{\mathrm{Fe}}^{2+}+H^{+}+{\mathrm{HOO}}^{•}$$

(3) $${\mathrm{Fe}}^{3+}{\mathrm{OH}}^{2+}\overset{\;\mathrm{hv}\;}{\to }{\mathrm{Fe}}^{2+}+{\mathrm{OH}}^{•}$$

The application of Fenton technology in the wastewater field has been more mature [12], but there are few studies on the treatment of VOC gasses by UV/Fenton technology, and no relevant literature on styrene degradation using UV/Fenton under nitrogen atmosphere has been found at present. Yoshinori Kawase et al. used UV/Fenton technology to degrade toluene gas and provided a promising path for the treatment of the volatile organic matter in waste gasses [10]. Lu í s M. Madeira et al. employed the Fenton process for up to 18 h degradation of toluene exhaust gas, which also removes by-products in the liquid phase while removing toluene in the gas stream [13]. Xiaolian Hu et al. compared the effect of Fenton’s reagent on COD in waste leachate with or without UV light irradiation and proved that the effect of UV/Fenton was stronger than that of Fenton [14]. In this study, a nitrogen gas stream containing styrene gas was formulated by blowing styrene with elevated purity nitrogen gas and bubbling in the liquid through the continuous air intake to study the effect of the UV/Fenton technique on styrene degradation under a nitrogen atmosphere. This study examined the effects of H_2_O_2_ concentration, Fe^2+^ concentration, pH and the optimal conditions [10]. Moreover, the possible degenerate products were analyzed. Finally, the obtained experimental data are taken as the sample set, and the BP neural network is improved by using the Longniushu algorithm to establish the BAS-BP neural network for the analysis of styrene gas degradation under different front conditions [15]. The BAS-BP optimization speed is higher than that of the BP neural network. The BP neural network was proposed by Rumelhard and McCleiand in 1986. It is a classical multilayer forward neural network. It consists mainly of two processes: forward propagation of the signal and backward propagation of the error. The forward propagating signal is processed by the neurons and then transmitted to the output layer. If the predicted value does not satisfy the accuracy requirement, the error is propagated. In this process, the error is assigned to each neuron, and the weights and thresholds are adjusted along the direction of the fastest downward adjustment of the error function until the output accuracy meets the requirements. Compared to the traditional machine learning (ML) algorithms, the BP neural networks have strong nonlinear mapping capabilities and are highly self-learning and adaptive. The Artificial Neural Network (ANN) used by Regina et al. trained the ML model to predict the ZnO optical band gap from experimental time and temperature conditions [16]. The best ML models they obtained were KRR and RR with quadratic features. The performance of these models is comparable to that of the effective mass models, and they generalize well to other datasets, thus preventing the effect of insufficient datasets on the simulation results. However, the training set of these models only includes two variables: time and temperature. The variables in this study are time, H_2_O_2_, Fe^2+^ and pH, and the accuracy of the model may not be guaranteed using traditional (ML) methods. This requires models to have a wider learning ability. In addition, the initial weight of traditional methods is randomly assigned, which may lead to the loss of model robustness, thus affecting the accuracy of the model. Therefore, we use BAS-BP models to find the weight corresponding to the global minimum of the loss function and update it iteratively on this basis. The BAS-BP neural network model in this study can judge the concentration of hydrogen peroxide in the solution from the simulation results as a judgment of the timing of the RE addition of hydrogen peroxide. It is also able to provide for the possibility of instrument failure inside the device when the actual results differ considerably from the simulated ones, with a view to providing a fresh pathway for the degradation of waste gas from the canning area. 

## 2. Materials and Methods

### 2.1. Chemicals

In this experiment, styrene (99.5%), ferrous sulfate (FeSO_4_·7H_2_O), potassium permanganate (0.01 mol/L) and sulfuric acid (98%) were all from McLean. Hydrogen peroxide (30% *w*/*v*) comes from Longda Chemical.

### 2.2. Experimental Procedures

**Figure 1:**
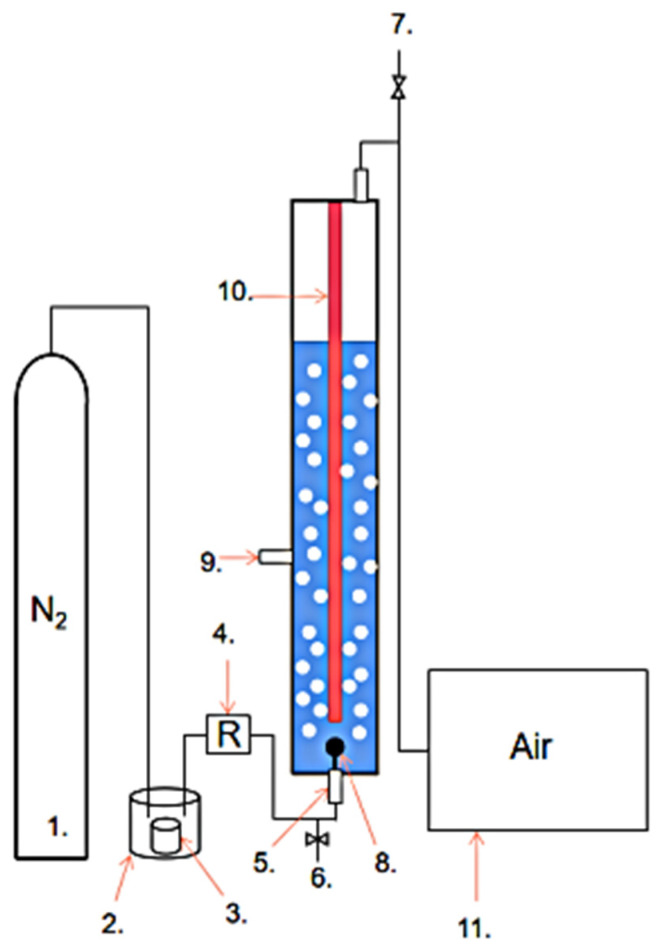
Device flow chart. 1. Nitrogen gas bottle 2. Wash bottle 3. Styrene vessel 4. Electron flowmeter 5. Air inlet 6. Front gas sampling port; 7. Back-end gas sampling port 8. Aerated stone 9. Liquid sampling port 10. Diving UV lamp 11. Gas chromatograph detector.

The experimental setup flow is shown in Figure 1. and elevated-purity nitrogen gas purged the styrene through a gas cylinder [17]. The concentration of styrene is adjusted by the height of the liquid level in the inner vessel of the wash cylinder. The reactor intake front is equipped with a gas flow meter, which is continuously supplied by varying the pressure of the nitrogen cylinder to control the gas flow rate at 3 L/min. The reactor was 1.4 m in height with an inner diameter of 10 cm and was made of quartz glass, which was equipped inside with a diving UV lamp of 40 W with one calyx [14]. The gas is continuously fed into the interior of the reactor through aerated stones.

### 2.3. Experimental Conditions

The control gas flow rate was kept constant at 3 L/min, the volume of solution in the reactor was 6 L, and the UV light intensity was 40 W. We explored the effect of different initial pH values (3.0, 5.0, 7.0), Fe^2+^ concentrations (0, 0.1, 0.3, 0.6 mmol/L), H_2_O_2_ concentrations (0, 10, 20, 30 mmol/L) on the degradation of styrene (2000 mg/L) gas [18]. In order to reduce experimental errors, two sets of parallel trials were performed at the same level for each influence factor. Due to the elevated contaminant concentration in this study, the experiment was terminated when the solution efficiency for styrene removal decreased to 50%.

### 2.4. Detection of DIOXYGEN Concentration

The hydrogen peroxide concentration was determined by potassium permanganate titration. A 25 mL sample of H_2_O_2_ was pipetted, placed in a conical flask, and 5 mL H_2_SO_4_ at a concentration of 3 mol/L was added, and the solution was titrated to light pink color with a standard titration solution of C (1/5 KMnO_4_) = 0.01 mol/L KMnO_4_, keeping it nonfading for 30 s.

The hydrogen peroxide concentration was calculated as follows:

(4) $$C_{H_{2}O_{2}}=\frac{C_{1}\times V\times 5÷2}{25}=V$$

Where—the concentration (0.5 mol/L) of C (1/5 KMnO_4_), *V*—the volume (ML) used for the titration

$C_{H_{2}O_{2}}$—hydrogen peroxide concentration (mmol/L)

### 2.5. Determination of Styrene Concentration (Determination of Treatment Efficiency)

Styrene concentrations before and after the reaction were determined using an East–West analytical GC-4000A meteorological chromatograph with a column box temperature of 90 °C, an injection port temperature of 150 °C, a detector temperature of 150 °C, a carrier gas of nitrogen, and an injection volume of 1 ml. It was obtained from a shift calculation of the xylene concentration in the gas phase before and after the reaction, i.e.,:

(5) $$\eta =\left[1-C_{out}/C_{in}\right]\times 100\%$$

In Formula η for xylene removal rate, $C_{in}$ and $C_{out}$ are the concentration of xylene in the inlet and outlet gases, mg/m^3^, respectively.

### 2.6. Determination of Liquid Phase By-Products TOC

The determination of total organic carbon was performed by combustion catalytic oxidation method at 680 °C with combustion oven temperature up to 700 °C. Experimental instruments included the use of a Tsushima TOC-V analyzer.

### 2.7. Characterization of Liquid Phase By-Products

Liquid by-products were measured with the Shimadzu GCMS-QP2020NX GC-MS/MS instrument. The splitless injection was used with a total flow of 30 mL/min, column flow of 1.08 mL/min, purge volume of 5.0 mL/min, and injection of 1 μL.

## 3. Results and Discussion

### 3.1. Determination of Optimal System

**Figure 2:**
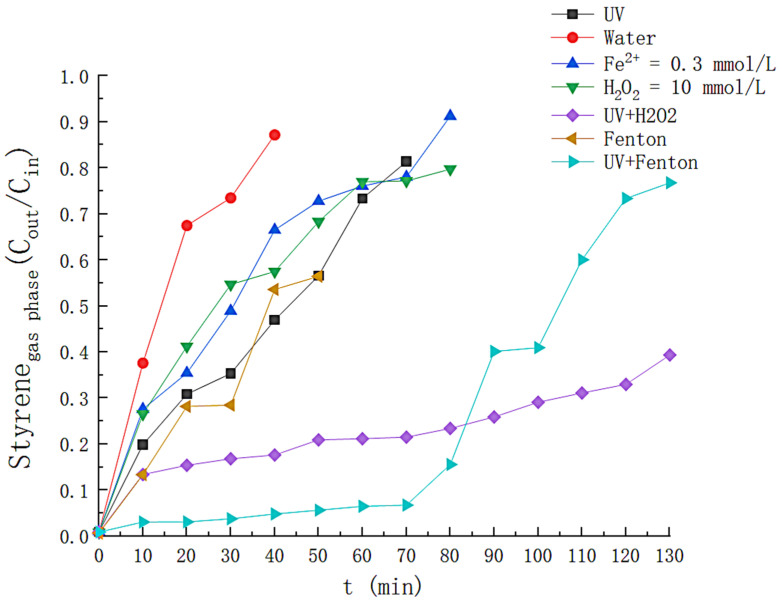
Experimental comparison of different reaction systems.

In order to explore the efficiency of styrene removal by different systems in nitrogen atmospheres, the time-dependent variation of styrene removal efficiency was examined for UV, Fe, H_2_O_2_, UV + H_2_O_2_, Fenton, and UV+Fenton conditions, respectively. The time-dependent profiles of the styrene gas removal efficiency for different conditions are shown in Figure 2. It can be seen that when only H_2_O, UV, Fe^2+^ and H_2_O_2_ are available, the styrene removal rate is the highest at the initial stage of the reaction and then decreases rapidly with time. The removal efficiency almost vanishes at around 60 min, suggesting that styrene is not yet degraded in the above system, and the reason for its removal is attributed to the adsorption of styrene gas by the liquid phase. The maximum removal efficiency of styrene gas by simple Fenton solution is 67%, which decreases to 17.5% at 50 min. The effect was not obvious because sufficient (•OH) for styrene degradation could not be generated under the condition of lower Fe^2+^ concentration. H_2_O_2_ can remove up to 86.6% styrene gas under the irradiation of UV light, and 40% styrene can still be removed at 130th min, because UV light could decompose H_2_O_2_ in tiny amounts, but the lack of catalyst in the solution led to the slow decomposition of hydrogen peroxide, and the degradation of styrene did not achieve the desired effect. Finally, the UV/Fenton system was employed to degrade styrene, and the solution exhibited an average removal efficiency of more than 90% for styrene within 70 min from the beginning of the reaction because the addition of UV light promoted the conversion between Fe^2+^ and Fe^2+^ and then increased the generation rate of (•OH) [10]. In summary, the UV/Fenton system shows the most significant effect on the degradation of styrene gas in nitrogen atmospheres, so the optimal operating parameters based on this system will be investigated.

### 3.2. Effect of Initial Solution pH on the Degradation of Styrene Gas by the Photo-Fenton Reaction

The solution pH can control the state of Fe^2+^ in solution and control hence the rate of hydroxyl radical production. The effect of pH on the results was tested under the following operating conditions: H_2_O_2_ = 10 mmol/L; Fe^2+^ = 0.3 mmol/L; UV = 40 W; gas flow rate = 3 L/min, and the solution volume = 6 L. The initial pH values were 3.0, 5.0, and 7.0, respectively (natural conditions).

**Figure 3:**
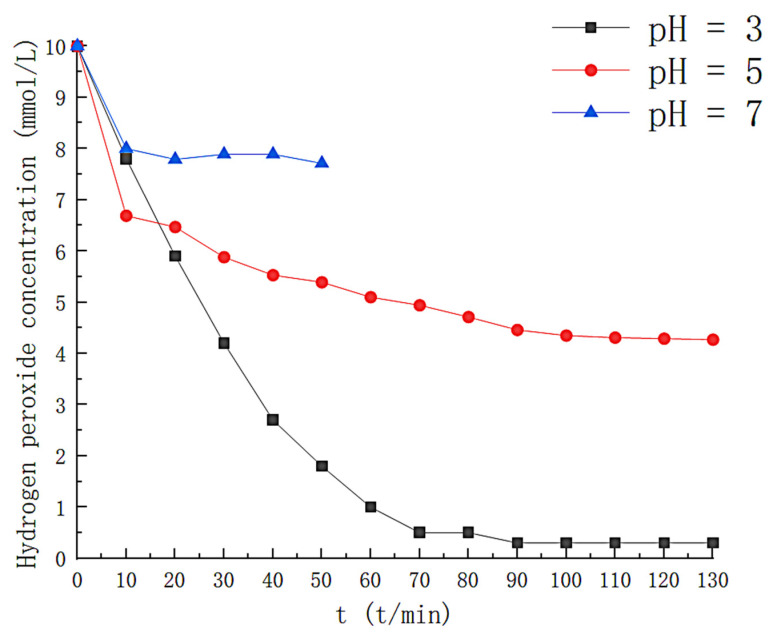
Effect of different pH on hydrogen peroxide.

**Figure 4:**
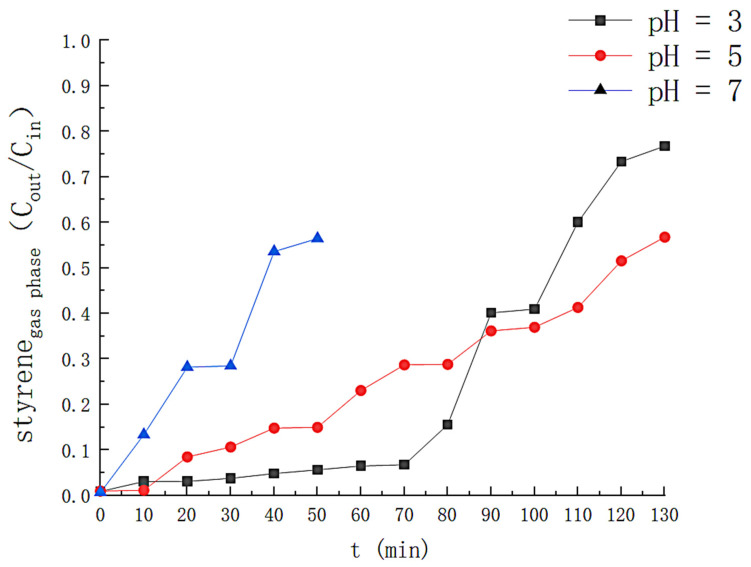
Effect of different pH on the removal efficiency of styrene.

**Figure 5:**
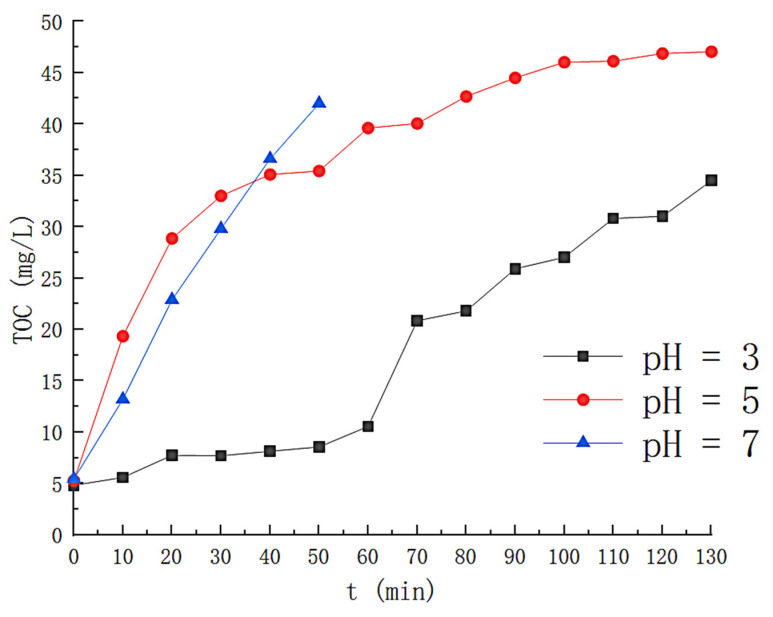
Effect of different pH on TOC concentration.

The effect of pH on H_2_O_2_ is shown in Figure 3, where at pH = 3.0, about 95% of H_2_O_2_ is consumed within 130 min, indicating elevated hydroxyl radical production in the solution. At pH values of 5.0 and 7.0, the maximum consumption of H_2_O_2_ was 57% and 23%, respectively, at times when the removal efficiency was higher than 50%. This is because the rise in pH increases the concentration of OH^−^ in the solution, which is unfavorable for the redox between Fe^2+^ and Fe^3+^, reducing the decomposition of H_2_O_2_. Figure 4 reflects the effect of pH on styrene degradation. For a pH of 3.0, the styrene removal efficiency is above 90% for the first 75 min and above 50% for 100 min; at a pH of 5.0, the styrene removal efficiency is higher than 90% in the first 20 minutes; at pH 7, the degradation effect of the solution on the styrene gas is significantly reduced, and the removal efficiency drops below 50% in about 35 min. At the same time, we also monitored the generation of intermediate products during the degradation process, and the results are shown in Figure 5. At pH 3.0, the TOC concentration in the solution rose slowly to 22.45 mg/L at 140 min after the reaction was initiated; at pH 5.0, the TOC concentration in the solution was 48.87 at the 120th min of the reaction, and at pH 7.0, the TOC concentration in the solution was 42.01. This is because the precipitation of iron with increasing pH was generated in the form of Fe[OH]_3_. The hydroxyl radicals that can participate in the degradation in the solution are reduced. Reducing the efficiency of styrene gas removal also increases TOC generation. Therefore, the optimal initial pH should be controlled at around 3. 

### 3.3. Effect of Fe^2 +^ Concentration on Styrene Degradation

The iron ions act as catalysts in the Fenton reaction, and the concentration of Fe^2+^ in the solution is varied by adjusting the concentration of iron salts from 0 to 0.6 mmol /L. The additional operating conditions were as follows: H_2_O_2_ = 10 mmol/L; pH = 3; UV = 40 W, and Gas flow rate = 3 L/min; solution volume = 6 L.

**Figure 6:**
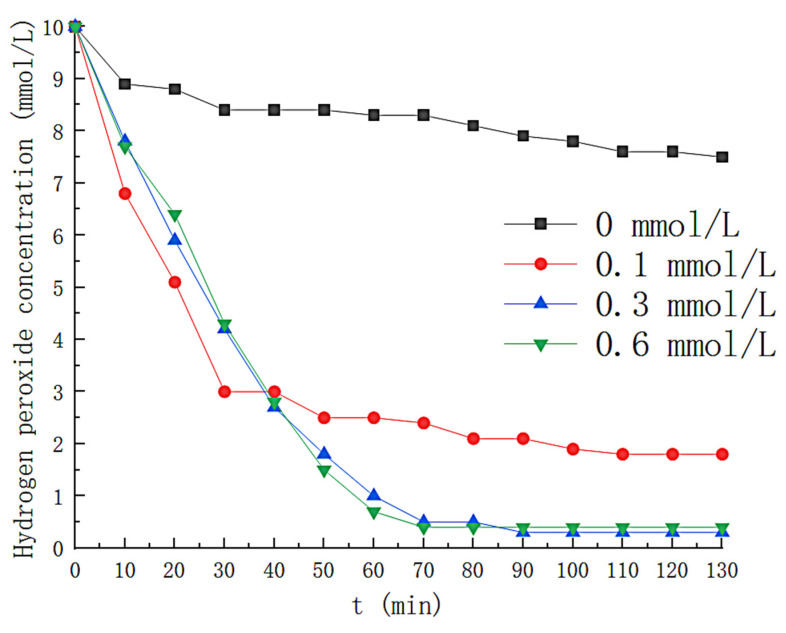
Effect of Fe^2+^ concentration on hydrogen peroxide concentration.

**Figure 7:**
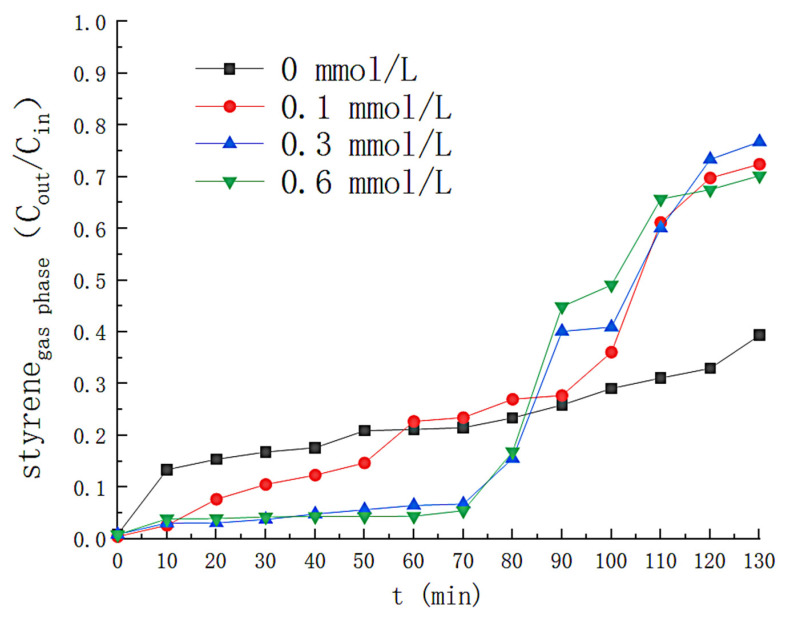
Effect of Fe^2+^ concentration on removal efficiency of styrene.

The effect of Fe^2+^ concentration on H_2_O_2_ is shown in Figure 6. The hydrogen peroxide consumption was slower without the addition of Fe^2+^, i.e., only a slight decomposition of H_2_O_2_ in the solution could not be quickly generated (•OH). The H_2_O_2_ in the solution failed to be fully consumed at Fe^2+^ concentrations of 0.1 mmol/L and at Fe^2+^ concentrations of 0.3 mmol/L and 0.6 mmol/L, respectively, with no significant difference in the rate of H_2_O_2_ consumption. The effect of Fe^2+^ concentration on the removal efficiency is shown in Figure 7, where the removal efficiency of styrene gas by solution increases with increasing Fe^2+^ concentration until Fe^2+^ concentration increases to 0.3 mmol/L. When the Fe^2+^ concentration was 0.3 mmol/L, the removal efficiency of styrene gas was above 90% in the first 74 min. The H_2_O_2_ concentration in the solution decreased rapidly during this period (shown in Figure 6), and H_2_O_2_ was nearly entirely consumed (about 95%) at the 70th min. After that, the Fe^2+^ concentration increased again, and the styrene removal efficiency did not alter significantly. After the 70th minute, because of the low concentration of hydrogen peroxide in the solution, Fe^2+^ could not continue to catalyze H_2_O_2_ production (•OH), resulting in a rapid decline in removal efficiency.

**Figure 8:**
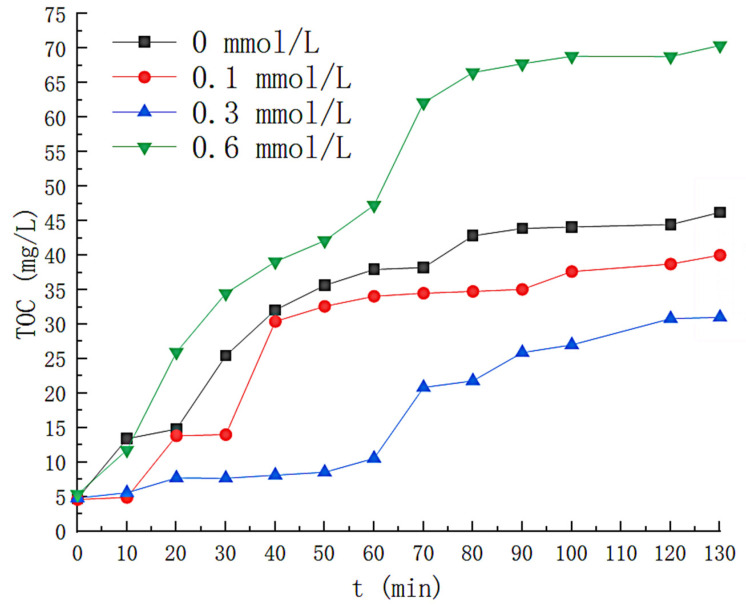
Effect of Fe^2+^ concentration on TOC concentration.

Under the front-end condition of Fe^2+^ concentration of 0.1 mmol/L, the efficiency of styrene removal by solution drops below 90% at the 20th min. The solution removed styrene with less than 40% efficiency when the reaction lasted for 110 min. This is because the catalyst concentration is not sufficient to fully decompose H_2_O_2_ under these conditions, thus reducing the removal efficiency. Figure 8 reflects the variation in the concentration of solution by-products. The lowest TOC concentration was produced in solution at a concentration of 0.3 milligrams per liter of Fe^2+^, which increased to 34.49 milligrams per liter when the reaction was continued for 130 min. The highest concentration of solution by-products was obtained at a Fe^2+^ concentration of 0.6 mmol/L, and the TOC concentration in the solution was as high as 70.41 mg/L as the reaction continued until 120 min. This is because the increase of Fe^2+^ promoted the generation of iron organic compounds. A large number of free radicals could not be produced to mineralize the reaction by-products when the concentration of Fe^2+^ was 0.1 mmol/L. Therefore, Fe^2+^ = 0.3 mmol/L is the best operating condition.

### 3.4. Effect of Hydrogen Peroxide Concentration

**Figure 9:**
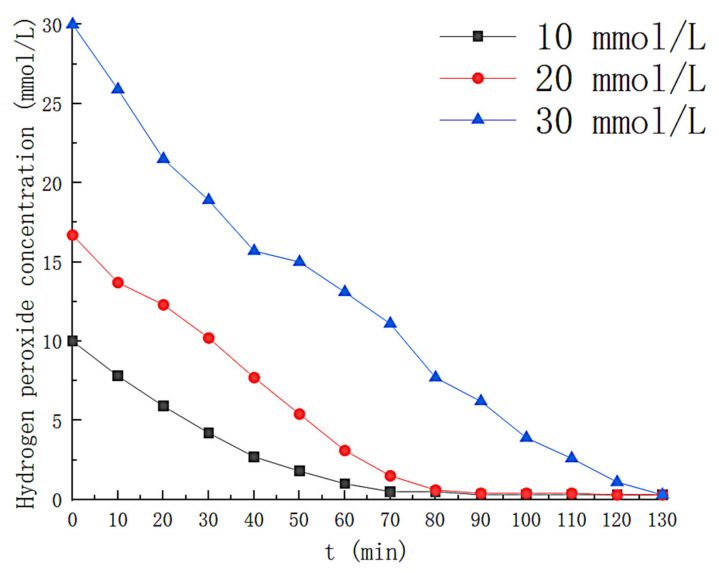
Change of hydrogen peroxide concentration with time.

**Figure 10:**
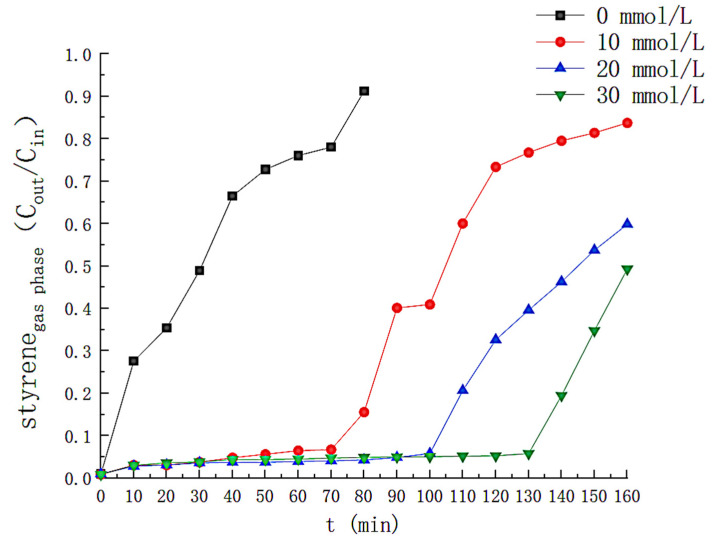
Effect of hydrogen peroxide concentration on removal efficiency of styrene.

**Figure 11:**
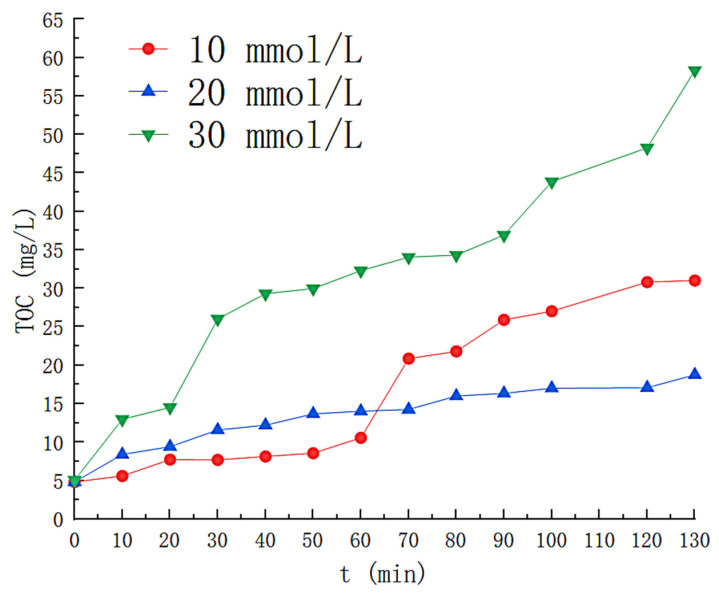
Effect of hydrogen peroxide concentration on TOC concentration.

Additional operating parameters were determined as follows: Fe^2+^ = 0.3 mmol/L; PH = 3; UV = 40 W, and gas flow rate = 3 L/min. The solution volume = 6 L. Hydrogen peroxide varies from 0 to 30 mmol/L. H_2_O_2_ is a source of (•OH), so H_2_O_2_ concentration affects (•OH) generation [11]. The variation of the H_2_O_2_ concentration with time is shown in Figure 9, where the concentration of H_2_O_2_ in the solution decreases rapidly for the first 70 min when the H_2_O_2_ concentration is 10 mmol/L, after which the H_2_O_2_ is almost entirely consumed(close to 0 mmol/L at 100th min). When the H_2_O_2_ concentration increased to 30 mmol/L, the time that the liquid phase contained H_2_O_2_ increased to 130 min. The effect of the H_2_O_2_ concentration on the styrene effect of the solution is illustrated in Figure 10. With H_2_O_2_ at concentration 0, the outgassing styrene content increases rapidly to near the front-end level. As the concentration of H_2_O_2_ increases, we find that it has a minor effect on the optimal removal efficiency but prolongs the removal time for styrene. When H_2_O_2_ was 10 mmol/L, the styrene removed by the solution remained above 90% for the first 75 min from the beginning of the reaction. The removal efficiency decreased rapidly thereafter, dropping to 14% at 160 min. When the H_2_O_2_ concentration was 20 mmol/L, the average styrene removal efficiency reached up to 96.2% within 105 min after the reaction started, and the outlet gas styrene concentration began to increase significantly at about 105 min and finally decreased below 50% at about 140th min. As the H_2_O_2_ concentration is increased to 30 mmol/L, its average removal efficiency for styrene is as high as 95.7% within 130 min. Figure 9 and Figure 10 reflect the effect of H_2_O_2_ in the UV/Fenton system, with the degradation effect rapidly decreasing as H_2_O_2_ is fully depleted. The variation in the concentration of the by-products in the solution is shown in Figure 11. The TOC content in the solution was the highest at the H_2_O_2_ concentration of 30 mmol/L and as high as 88.79 mg/L at 130 min. When the concentration of the H_2_O_2_ was 10 mmol/L, the first 60 min TOC content was less than that of the solution with the H_2_O_2_ concentration of 20 mmol/L, but thereafter the TOC content in the solution with the H_2_O_2_ concentration of 20 mmol/L grew slowly. The final H_2_O_2_ concentration of 20 mmol/L solution showed a TOC of 22.45 mg/L at 140th min, less than 34.49 mg/L of 10 mmol/L. The efficiency of removal of styrene gas by different concentrations of H_2_O_2_ in this study is less varied. The effective removal time of the solution for styrene (removal efficiency greater than 90%) was not exponentially prolonged as the H_2_O_2_ exponentially increased. An H_2_O_2_ concentration of 20 mmol/L was chosen as the optimal concentration for photo-Fenton degradation of styrene gas by H_2_O_2_ in a nitrogen atmosphere, taking into account industrial economic benefits and by-product production.

## 4. Mechanistic Analysis

**Figure 12:**
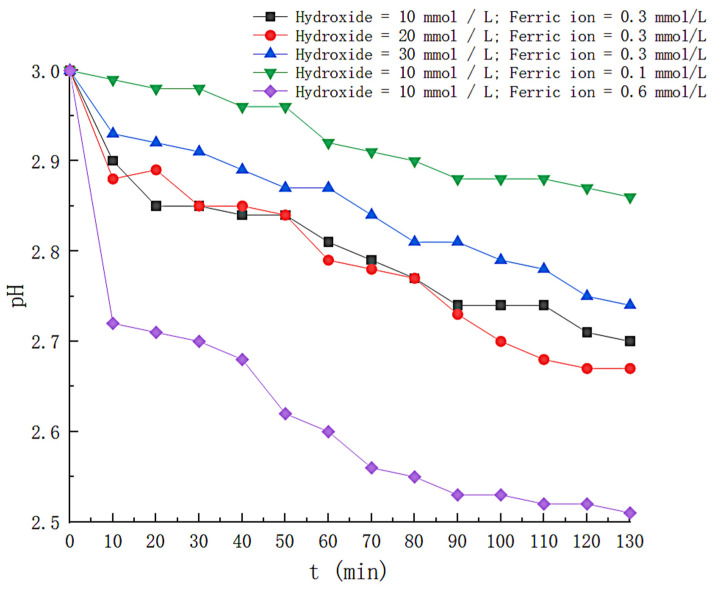
Variation of pH under different conditions.

**Figure 13:**
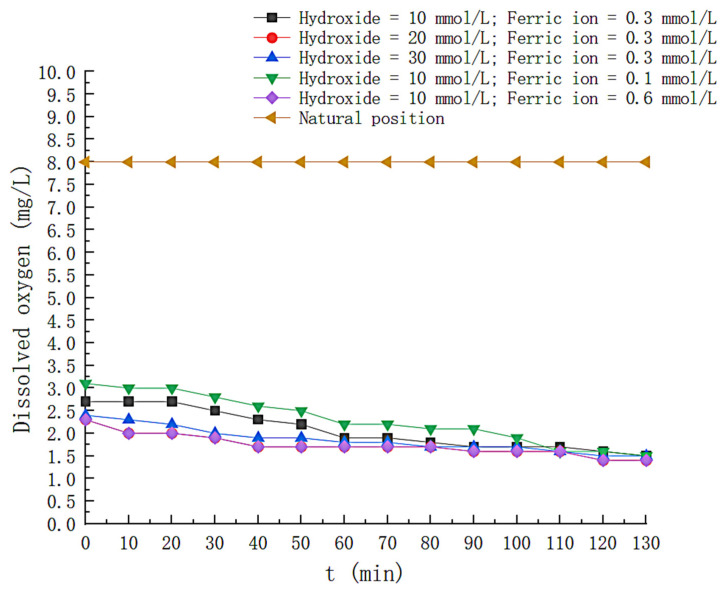
Variation of dissolved oxygen under different conditions.

The shift of the pH value during the experiment is monitored, as shown in Figure 12. The pH values of the solutions under different conditions all show a decreasing trend with time. Luis M. Madeira et al. mentioned in the literature that this is because some organic acids (maleic acid, pyruvic acid and oxalic acid) are contained in the reaction by-products [19]. In order to distinguish nitrogen gas bubbling from air bubbling, the time-dependent shift of the dissolved oxygen content in the solution was recorded, and the results are shown in Figure 13. Nitrogen as a carrier gas decreases the concentration of oxygen in the solution, and the concentration of dissolved oxygen during the experiment was floating at 2.0 mg/L, considerably less than 9 mg/L in normal water.

**Figure 14:**
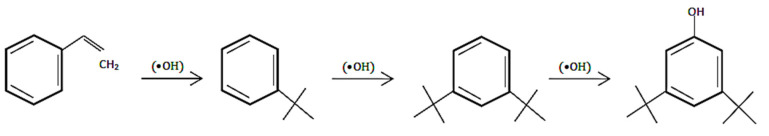
Reaction mechanism of styrene.

Qualitative analysis of the final liquid phase by-products was carried out by gas chromatography-mass spectrometry. It was observed that there were many carbon long-chain organics in the liquid phase, among which 3,5-di-tert-butylphenol was the most abundant. The reaction mechanism of styrene forming by-products under the action of (•OH) is shown in Figure 14.

## 5. BAS-BP Neural Network Model

The beetle antennae search (BAS), also called the crustacean algorithm, is a novel algorithm inspired by the foraging principle of the cow [15]. According to the biological rationale, when the longhorn beetle is foraging, it doesn’t know where the food is, but it focuses on the food by its smell. The longhorn beetle has two antennae, and when the left antenna collects an odor stronger than its right, it flies to the left and vice versa. According to this method, the cows can quickly find food. The advantages of BAS are that only a single individual with a single function needs to be found, an automated optimization process can be achieved without specific functions, and a reduced number of iterations leads to a significant improvement in the speed of optimization [20].

### 5.1. BAS-BP Neural Network Model Building

The right-hand side and threshold values of the BP neural network are optimized using BAS, which is modeled with the following specific steps:

(1) Normalization was performed on the random vector of calf whiskers:

(6) $$\vec{b}=\frac{rands\left(k,1\right)}{\left\|rands\left(k,1\right)\right\|}$$

Where $\vec{b}$ is a random variable with the day cow whisker orientation; rands () as random function; K represents the spatial dimension.

(2) Creating apart left and right whisker spatial coordinates:

(7) $$\begin{array}{l} x_{rt}=x^{t}+d_{0}\times \vec{b}/2\\ x_{lt}=x^{t}-d_{0}\times \vec{b}/2 \end{array}$$

Where $x_{rt}$ is the position coordinate of the day bovine left whisker at iteration t; $x_{lt}$ is the position coordinate of the day bovine right whisker at iteration t; $x^{t}$ is the coordinate of the center of mass at the iteration of day bovine t, and $d_{0}$ represents the distance between the two whiskers of the day cow.

(3) The odor intensities of the left and right whiskers of day cattle, i.e., the intensities of $f\left(x_{r}\right)$ and $f\left(x_{l}\right)$, were expressed according to the adaptation function f().

(4) The direction of movement of the position of the cow was judged according to the odor intensity:

(8) $$x^{t+1}=x^{t}-\delta^{t}\times sign\left(f\left(x_{rt}\right)-f\left(x_{lt}\right)\right)$$

where $\delta^{t}$ represents the step factor at the tst iteration; sign() as a symbolic function.

(5) Let the step factor be given. Due to its ability to control the area searchability of the calf’s whisker, a step factor as large as possible was chosen so that it covered the search area sufficiently to not get trapped in a local minimum:

(9) $$\delta^{t+1}=\delta^{t}\times eta\;t=\left(0,1,2\cdots n\right)$$

(6) The root means the square error MES of the test sample is taken as the fitness evaluation function:

(10) $$fitness=MES=\frac{1}{N}{\sum_{i=1}^{N}\left(t_{sim\left(i\right)}-y_{i}\right)}^{2}$$

where fitness is the fitness evaluation function value; N is the number of training set samples; $t_{sim\left(i\right)}$ is the ith sample model output value, and $y_{i}$ is the actual value of the ith sample. The minimum of MES at the iteration stop is the optimal solution we want.

**Figure 15:**
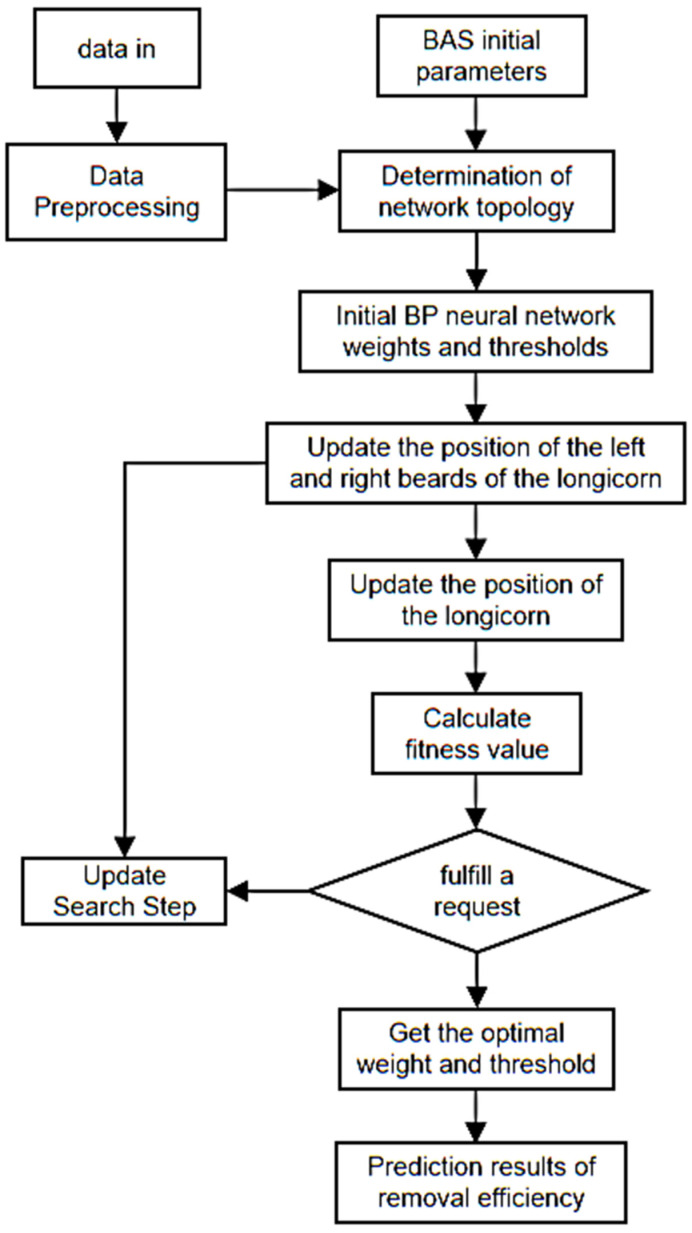
BAS optimization process of BP neural network.

(7) Calf position was initialized. The initial position of the cow was then given and saved in bestX.

(8) Fitness functions were calculated for the initial positions and saved in bestY.

(9) Update the calf left and right whisker position coordinates according to Formula (2).

(10) Values of $f\left(x_{r}\right)$ and $f\left(x_{l}\right)$ were taken on the basis of the position of the left and right whiskers in the cows. Update the fitness function value according to Equation (3) after comparing the intensities, and update bestX and bestY if the value f() is better than bestY.

(11) The algorithm ends the run when the number of iterations reaches 100.

(12) The solution bestX when stopping the iteration is the trained optimal solution. That is, the optimal right and threshold value of the BP neural network: after it is taken into the neural network, the BAS-BP neural network model is finally generated. The neural network structure model was built according to the above conditions. The results are shown in Figure 15:

### 5.2. Results and Discussion

In this paper, a total of 17 sets of experimental data are selected as the total sample, using the previously mentioned experimental data as the training set, varying the front-end conditions, and four sets of calibration tests are used as the test set, using MATLAB 2021b as the platform.

**Figure 16:**
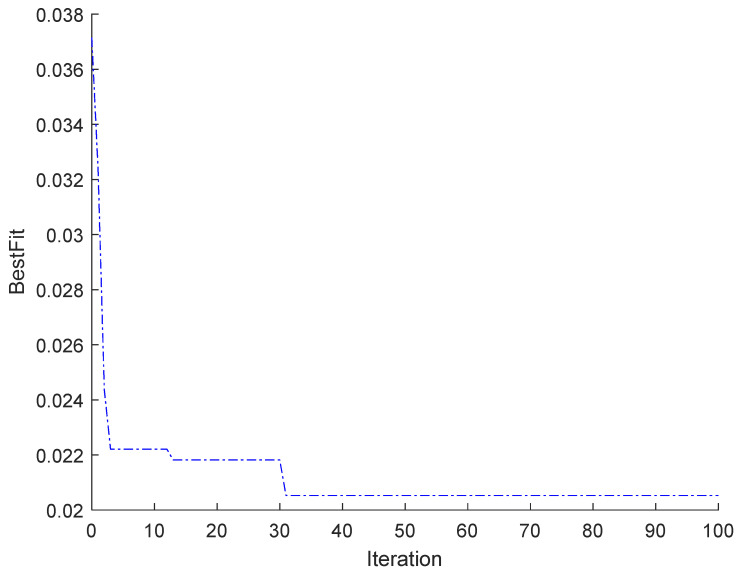
Fitness curve of BAS-BP neural network.

**Figure 17:**
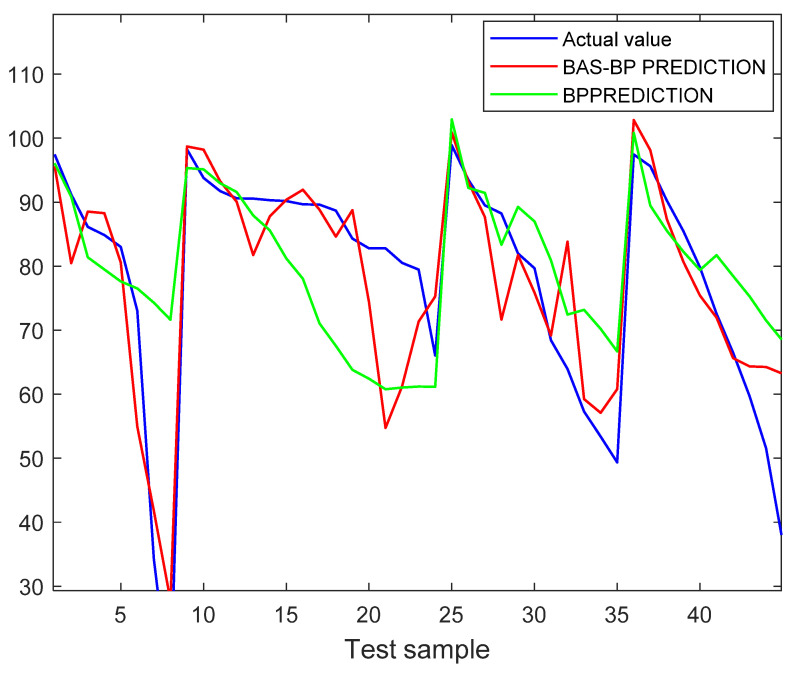
Fitting results of the training set of BAS-BP neural network.

Figure 16 shows the iterative results of the optimization of the bas algorithm. It is known from the figure that approximately 31 iterations are close to convergence, at which point the result is the closest to the best fit. That is, the optimal solution is 0.0205259 and 31. Figure 17 shows the results of the fitting of the BAS-BP with the BP training set, and it can be seen from the figure that the predicted results and the curve trends of the optimized BAS model are close to the true values.

To realize the optimization effect of BAS on BP, Table 1 lists the mean square error (MSE), root mean square error (RMSE) and mean absolute error (MAPE) as evaluation indicators for model prediction [18], and the results are shown in Table 1: the BAS-BP decreased by 64.22%, 40.19% and 54.21%, respectively, compared with the BP indicators. The above results indicate that the BP neural network after BAS optimization has a more pronounced improvement in terms of convergence rate or accuracy.

## 6. Consequent

The degradation of styrene in a nitrogen atmosphere by Fenton’s reagent in a bubbling reactor was investigated. Parametric studies were carried out, and the following conclusions were obtained: The UV/Fenton process showed a favorable effect on styrene degradation under the nitrogen atmosphere. The styrene effective removal time increased with the increase of H_2_O_2_ concentration, and when H_2_O_2_ in the solution was fully consumed, the styrene removal efficiency showed an obvious decrease. The optimum conditions for styrene degradation were obtained at the H_2_O_2_ concentration of 20 mmol/L, a Fe^2+^ concentration of 0.3 mmol/L, and a pH of 3, which could degrade styrene gas at a concentration of 2000 mg/L with 96.2% efficiency within 100 min and a final by-product (TOC) concentration of 22.45 mg/L. In addition, the established BAS-BP neural network model performs better than BP neural network and can be evaluated for styrene removal efficiency with different parameters. This model can provide a series of valuable data under some front-end conditions that are difficult to operate. The reference provides real-time data for certain difficult-to-monitor quantities such as hydrogen peroxide content, TOC concentration, etc. The oxidant content in the reactor can also be back extrapolated by simulation results, which guarantees that the device can operate for a lengthy time. It is capable of alerting the operator to the presence of instrument damage within the reactor in some cases where the actual and simulated data do not correspond.

## Figures and Tables

**Table 1:** Effect comparison of different models.

Algorithm Name	MSE	RMSE	MAPE
BAS-BP	90.6499	9.521	11.8578
BP	253.3777	15.9178	25.8973

## Data Availability

Data sharing is not applicable; no new data were created or analyzed.
